# How health care professionals handle limited resources in primary care – an interview study

**DOI:** 10.1186/s12913-022-08996-y

**Published:** 2023-01-03

**Authors:** Suzana Holmér, Ann- Charlotte Nedlund, Kristin Thomas, Barbro Krevers

**Affiliations:** 1 Department of Health, Medicine and Caring Sciences, Linköping University, Sandbäcksgatan 7, 581 83 Linköping, Sweden; 2 Department of Health, Medicine and Caring Sciences, Swedish National Centre for Priority Setting in Health Care, Linköping University, Linköping, Sweden

**Keywords:** Health priorities, Priority setting, Ethical principles, Primary health care, Rationing, Qualitative methods

## Abstract

**Introduction:**

Health care systems around the world are struggling with limited resources, in relation to the prevailing health care need. An accessible primary care is an important part of the solution for how to provide affordable care for the population and reduce pressure on the overall health care system such as unnecessary hospital stays and associated costs. As primary care constitutes an important first line of healthcare, the task of prioritising and deciding what to do and for whom lies in practice, primarily with the primary care professionals. Thus, the decisions and behaviour of primary care professionals have a central role in achieving good and equal health in the population. The aim of this study is to explore how primary health care professionals handle situations with limited resources and enhance our knowledge of priorities in practice.

**Methods:**

Semi-structured interviews with 14 health care professionals (7 nurses, 7 physicians) working in Swedish primary care were interviewed. Data were analysed inductively with content analysis.

**Findings:**

Three main categories were found: Influx of patients; Structural conditions; and Actions. Each category illustrates an important aspect for what primary care professionals do to achieve good and equal care. The influx of patients concerned what the professionals handled in terms of patients’ healthcare needs and patient behaviour. Structural conditions consisted of policies and goals set for primary care, competence availability, technical systems, and organisational culture. To handle situations due to limited resources, professionals performed different actions: matching health care needs with professionals’ competency, defining care needs to suit booking systems appointments, giving care at the inappropriate health care level, rearranging workhours, and passing on the decision making.

**Conclusion:**

Priorities in primary care are not, “one fits all” solution. Our study shows that priorities in primary care comprise of ongoing daily processes that are adapted to the situation, context of patient influx, and structural conditions. Healthcare professional’s actions for how influx of patients’ is handled in relation to limited resources, are created, and shaped within this context which also sets the boundaries for their actions.

## Introduction

In many countries primary care is often seen as an important part of the solution for how to provide affordable and accessible care for the population and reduce the pressure on the overall health care system, such as unnecessary hospital stays and associated costs [1–4]. Consequently, primary care has gained extensive responsibilities and high expectations of providing a wide range of care services for the population in terms of outpatient care, such as examinations, diagnoses, treatments, but also rehabilitation, disease prevention, health promotion, and patient education [5, 6].

Health care systems around the world are struggling with limited resources, in relation to the prevailing health care need [7, 8]. That puts focus on the question of how to prioritise and allocate available resources. This means not only to prioritise and allocate pure monetary resources but also resources in terms of e.g., personnel, competences, equipment, pharmaceuticals, facilities. Furthermore, as publicly funded health care systems depend on citizens’ trust, and willingness to contribute to the common welfare through taxes, it is also of importance that citizens perceive the allocation of resources as fair [9]. A few countries, for example Sweden and Norway, have formulated ethical principles that are regulated in law for how to prioritise and allocate resources in health care fairly as a support for decision makers and clinician.

Primary care is an important first line of healthcare to which patients are expected to primarily seek care for, a wide variety of health problems. This put demands on the healthcare staff to make the necessary priorities on an individual level and between different patient needs.

Although there are ethical principles for potential guidance, primary care professionals report difficulties to decide how to prioritise and distribute care [10–12]. One reason for this is that professionals find it hard to balance multiple demands from patients, co-workers, and the organisation as they have dual roles: one as a carer with responsibilities towards the patients [13] and, one as a gatekeeper of limited services [13, 14].

Political governance on primary care, such as reforms on accessibility [15] and various financial compensation models [16, 17], may limit professionals’ possibilities to explicit prioritisation in accordance with how these decisions ethically should be done stipulated in law. In Sweden, patient choice of primary care implies that the primary care centres receive a fixed fee by the region for each listed individual [16, 17]. Professionals must therefore simultaneously take responsibility for available resources by prioritising, without making the patients dissatisfied so they choose another primary care centre instead [18]. Additionally, professionals could consider that their professional responsibility for the individual patient makes it hard to deny or ration care [18–20].

At the end of the day, everyone must prioritise to solve situations with limited resources. Previous studies in primary care regarding professionals' views on priorities, rationing, and related ethical principles, have often used fictional scenarios or questionnaires [19, 21, 22].

However, such methods do not show how prioritisation is done in their everyday practice [22], and the knowledge through research is still limited on these issues. There is a need for inductive studies to yield insights into what and how the primary care professionals do in practice, to allocate resources to meet the patients’ healthcare needs.

The aim of this study is to explore how primary health care professionals handle situations with limited resources and enhance our knowledge of priorities in practice.

### The Swedish health care context

The present study is conducted in Sweden where priority setting and resource distribution in health care, should follow three ethical principles in accordance with the Swedish Parliament’s decision from April 1997 [23]. The ethical principles are: *the human dignity principle*, which is the overriding ethical principle that all people have equal value and the same right to care, regardless of personal characteristics, and functions in society; *the need-solidarity principle*, which implies that those in greater need, with more severe diseases, and worse quality of life should be given priority in distributing healthcare resources; and *the cost-effective principle*, which means that there should be a reasonable relation between the cost and effects of a treatment. Furthermore, the ethical principles are included in the goal of the Swedish Health and Medical Services Act [24], which states that the authorities, county councils and municipalities are responsible to strive for good and equal health in the population and provide health care on equal terms [24].

In Sweden, the county councils provide primary care and secondary health care at hospitals, while municipalities provide home health care and nursing home care. Furthermore, in these homes, the first medical assessments are carried out by specialist nurses employed by the municipalities, while the county council employed primary care physicians, are consulted when needed [25]. In Sweden, primary health care centres are multi-professional. Beyond physicians, registered, specialist- and assistant nurse, allied health care professionals such as, psychologists, dieticians, and physio- and occupational therapists, also work there. They are either located at the centres, or in another organisation within primary care, such as childcare, maternity care, or rehabilitation centres.

However, to get an appointment to a primary care professional, citizens can contact primary care centres via telephone or through digital services. Their medical concern is assessed by appropriate health care professional, usually by nurses. So called “walk-ins”, when patients seek care in person and without any previous assessment of care need, is not encouraged.

## Method

This study is a qualitative interview explorative study using inductive content analysis [26]. Data is based on individual interviews with health care professionals (nurses and physicians) working in primary care. The consolidated criteria for reporting qualitative research have been met to increase the quality of reporting [27].

### Study setting

The study was conducted in a county council in the southeast of Sweden, with approximately 450,000 inhabitants. About 360,000 are listed as patients in the 33 public primary care centres, that are administrated by the county council’s office for primary care. The remaining inhabitants are either listed at county council-funded privately owned centres, private alternatives or unlisted.

### Recruitment procedure

A purposive sampling was used to achieve a varied sample of primary health care centres from which informants were recruited. Four of the 33 primary care centres were selected based on location (urban/rural), size, in terms of the number of listed patients and the number of physicians in relation to patients, with the intention of gaining a variety of different situations that might occur due to limited resources. Two centres had rural locations with approximately 7000 and 14,000 listed patients, respectively. The other two centres provided care in urban areas and had approximately 20,000 listed patients each. Two of the primary care centres, one in a rural area and one in an urban area, were considered to have few physicians in relation to number of patients.

For our study we invited physicians and nurses, with varied specialities and competence levels. Staff that held managerial positions such as, operational managers were not included, as their responsibilities and the content of their duties in the organisation did not suit our aim.

The recruitment was carried out in four steps: Step 1) the head of the country council’s office for primary care was informed about the study and gave permission to carry on the recruitment and conducting interviews during professionals working hours; Step 2) the operational managers of the four selected primary care centres then informed about the study. They all gave permission, and supported the recruitment process by providing access to staff meetings and email lists; Step 3) individuals who fitted the inclusion criteria, were informed about the study, and invited via e-mail and staff meetings to participate in the study; Step 4) individuals who showed interest were asked to answer a questionnaire with six questions regarding demographic data (gender, age, profession, years of clinical work, years of clinical work in primary care, and current workplace). The questionnaire provided demographic information about the interested individuals, and through this we were able to ensure that the inclusion criteria were met. All communication with the informants regarding the study, and scheduling of interviews, were carried out via e-mail and staff meetings.

### Informants

In total 14 informants (7 women and 7 men) were interviewed. Nurses included registered and specialist nurses who had worked between 3- 42 years in health care and between 4 months- 26 years in primary health care and all were women. Physicians included medical interns and general practitioners who had worked between 1.5—44 years in health care and 4 months- 35 years in primary health care and all physicians except one were male. All informants were permanent employed, except for two medical interns who were employed for at least six months.

### Data collection

A semi-structured interview guide was developed by the research team based on the study purpose. The team includes a doctoral student (first author) and three senior researchers with extensive experience in qualitative health care research. Two of the researchers (SH1; BK4) have a background as registered healthcare professionals who performed clinical work, one of which in primary care. The interview guide contained two main questions: a) How do you handle situations with limited resources in your everyday work? b) How is the collegial collaboration at your workplace? In addition, encouraging follow-up questions and probes were used when needed, such as, can you explain a little more, what happens and when, in what situations, what do you do, what do your colleagues do. The guide was initially tested in a pilot interview, which was included in the analysis, and no significant changes were made to the interview guide. The interviews were conducted during work hours in locations chosen by the informants. Interviews lasted about one hour (range 41- 78 min). The interviews were conducted by the first author (SH1) and then audio-recorded and transcribed verbatim by a professional contractor. One recording failed due to a technical malfunction, that interview was instantly documented through written notes, which were included in the analysis.

### Data analysis

The transcribed text was analysed using qualitative inductive content analysis [26]. The first author (SH1) became familiarised with the data by reading the transcripts several times. Text relevant for the study aim was highlighted and coded using labels that described its content. These initial codes were documented in a code book and primarily organised in categories where codes with a common pattern created a category. The fourth author (BK4) also read the transcripts and discussed the code book and categorisation with the first author. Furthermore, the categories and text were revisited in an iterative process with all authors, to ensure the trustworthiness of the analysis.

### Ethical approval

According to Swedish legislation on ethics in research on humans [28], ethical approval was not needed as the study did not include any intervention or sensitive data. The ethics of the study was guided in accordance with the World Medical Association’s Declaration of Helsinki [29]. All informants received written information about the study, and their rights stipulating that their participation was voluntary and that they were free to withdraw at any time. They were assured of de-identification and confidentiality, and this information was repeated verbally before the start of interview. Furthermore, they were given the opportunity to ask questions about the study before signing the consent form. The consent form was signed before being interviewed.

### Findings

The analysis led to three main categories describing how professionals handle situations with limited resources (Table 1). The findings illustrate the multifaceted circumstances that the staff meet every day and that must be handled. The first category presented is, influx of patients, which describes what professionals handled, and how it affected prioritisation decisions. The second category, structural conditions, describes prerequisites for what could be done. The third category, actions, describes how professionals handled situations with limited resources.

**Table 1:** Handling situations with limited resources

*Category*	**INFLUX OF PATIENTS**	**STRUCTURAL CONDITIONS**	**ACTIONS**
*Sub-category*	Variation in health care needs	Policies and goals	Matching health care needs with professional’s competency
	Patient behaviour	Competence availability	Categorising health care needs to suit appointment booking systems
		Technical systems	Giving care at the inappropriate health care level
		Organisational culture	Rearranging workhours
			Passing on the decision making

## Influx of patients

Primary care was described as having an important role for both the population and the individual patient, as it was the first line of care. The informants described that the primary care centre, by being the main provider of healthcare in the community, provided both a broad range of services and offered a sense of safety in that specific geographical area. Although it was stated that many patients contacted primary care, it was mainly aspects involving their varied health care needs, behaviour, decisions, and legal right to choose a caregiver that were eventually handled. The informants described that the influx of patients varied between weekdays. More patients sought care Mondays compared to Fridays, (as primary care centres did not operate during weekends).

“…Thursdays and Fridays’ are usually quieter on the phone, but yes, Mondays are the worst…”-Nurse 4

Patients were described having multiple options for how to contact their primary care centre, such as, calling the national health advice services, using digital services, or writing a letter to the centre. Furthermore, patients were also referred by other health care workers, usually from hospital wards. Language restrictions, vicinity to the primary care, urgency, not getting hold of a nurse or dissatisfaction with the telephone assessment, were some reasons listed by the informants for why some patients sought care directly at the centre. Despite many contact options, the informants emphasised that most patients chose to call the primary centre’s telephone to talk with a nurse.

### Variation in care needs

Health care needs varied between patients. The informants described health care needs in terms of, care consumption, urgency of care, extent of care but also, in relation to demand on the treating professionals.

“I think about those with multiple diseases, they have a lot, they have leg ulcers that they get care for//they have chronic obstructive pulmonary disease, then there is their age, they are multi-sick…” -Nurse 5

When quantifying “how much care” was needed for a patient, the informants described it in terms of, greater or minor health care needs. For example, patients with multiple chronical diseases, memory problems or psychiatric diagnoses, were described as having great health care needs, and needing more care. In contrast, patients that were normally healthy but suddenly deteriorated with an infection, were exemplified as having minor health care needs. Even minor health conditions were described as urgent and sometimes requiring immediate care. One informant explained how a patient with a small cut from a kitchen knife, needed immediate plastering to prevent an infection, even if the cut itself was not considered “great”.

Additionally, interviewed physicians described health care needs from a professional standpoint, referring to demands on the professional- how difficult or easy the issue is for them to examine or treat. Patients with neuropsychiatric disorders requesting unnecessary or harmful measures were given as an example of difficult health care needs, as good communication skills were needed.

As the primary care centre was the first instance for many patients, the informants also handled “patients” with needs and measures that were outside publicly funded health care, such as community-based needs.

“…there is perhaps a mental illness or relationship problems or things like that, that they do not want to reveal through the phone, well it is not always that they say how it is through the phone, they have either exaggerated in order to get an appointment, or it is something else entirely. So, it is hard for the nurses, I do not know what they [the patients] said through the phone, maybe it was a correct assessment of that situation, but, actually, it was not that dreadful…” -Physician 4

The common way patients contacted the primary care centre was by calling the phoneline, where they were able to talk to a nurse who evaluated their symptoms. According to the informants, evaluating symptoms through the phone was difficult, and multiple reasons were described for why the nurses’ ability was challenged. Informants explained that some patients exaggerated symptoms to get booked for an appointment, while others downplayed their issues, to not be advised to go to the emergency care. As explained by one informant, a patient’s chest pain could be a potential heart attack which cannot wait. Another reason was, that patients did not always want to disclose their real cause for contact through the phone. Patients suffering from mental illness or had relationship problems, were two examples of when the patient did not always disclose how it is*.* Furthermore, the informants did emphasize an understanding that resources were sometimes wrongfully used, as patients were booked to a professional with a higher or lower competence level, required for the patients’ health concern.

### Patient behaviour

Interviewed physicians explained that patient behaviour contributed to which prioritisations were made. One informant explained how a patient asked many questions during their appointment, which left little time for the actual medical examination. This behaviour forced the informant to choose between examinations, all of which could have been managed during this one appointment.

“Because I think that those who have chosen to work within health care have a desire to help and please people and help them as best they can. But, if someone is half an hour late when I have a fully booked schedule, then the feeling for them is not comparable to the need that other patients have… so in those case one has to cancel [the next appointment]” -Physician 5

Furthermore, one physician explained how patients’ behaviour, such as being late for an appointment, forced them to prioritise between patients, which could otherwise have been avoided. As the physicians’ schedules are usually fully booked, the late patients- or the following patients’ appointments, must be cancelled or shortened. The physician stated that the prioritisation in these situations was individual and depended on how much work was waiting to be done, and how nice they could be.

According to the informants, patients’ behaviour and actions were unforeseen, which forced them to indirectly prioritise patients for an examination. As previously explained, patients who could not get hold of a nurse or were dissatisfied with the medical assessment through the phone, sought care directly at the primary care reception. These patients had considerably varied health care needs, such as chest pain, cuts, or ingrown nails. An interviewed nurse explained that these so-called “walk-ins” were examined the same day, and hence, indirectly displaced health care needs that were considerably greater, and should have been prioritised. Nevertheless, the informants had experience with how patients care search pattern could be influenced. According to one interviewed physician, patients´ expectancies regarding antibiotics prescriptions was altered, when a new public recommendation for otitis switched, from a liberal prescription to more restricted use. Thus, formal guidelines could play an important role in changing patient behaviour.

## Structural conditions

To perform different actions, the informants described structural conditions that were important for what they could do, and how they would do it. These conditions are described through the four subcategories.

### Policies and goals

According to the informants, policies and goals for primary care which are set at both the national and county council levels, were affecting what was eventually prioritised by health care professionals. The informants elaborated that the law stipulating a waiting time of maximum three days for an appointment in primary care, was not always of medical relevance, and restricted their ability to de prioritise patients with minor health care needs.

“it can have to do with continuity or somebody that I had contact with a long time ago, that I consider will after all get the best help from me, not meet somebody new. So, you could say that continuity is a part of prioritisation” -Physician 4

According to the informants, the policy for continuity of care, was important for some patients and not others. Compared to minor health care needs, continuity was expressed as a part of prioritisation for patients with greater health care needs. Hence, it was explained as beneficial for patients with complex diseases, comorbidity, and a long list of drugs, to meet the same physician. The informants explained how finding a balance between treatments and their possible side effects, was a *“delicate job”(*Physician 1), and therefore it was better for these patients to meet the same physicians. The informants emphasised that the availability of personnel varied, depending on unforeseen things, such as sick- and parental leave, and therefore maintaining continuity, and patient centredness was sometimes a concern for patients with greater health care needs.

The informants described with indignation how political decisions contributed to, that the healthcare changed in a direction where the resources for those with greater healthcare needs risked being used by those with minor needs. An example of this was the county council's decision to start a digital primary care center where patients could book an appointment directly with a doctor without prior assessment by a nurse. The informants believed that those with mild conditions would be favored by this.

### Competence availability

In structural conditions, availability of health care competencies, is included, which individual professionals were scheduled to work, and which time slots were available for booking. According to the informants, the lack of appropriate competencies created situations where prioritisation was needed. For example, lack of appropriate competence needed to perform a procedure, directly lead to patients being de-prioritised. To be able to perform care availability to examination rooms is needed and could also limit how different competencies were used.

”But then if a nail thigh is to be operated on, then you must have an assistant nurse available too, which can help, so that is another component on the whole, or prioritisation, how it is [the availability]” -Nurse 1

The lack of available time slots created a stressful work environment. The interviewed nurses described that they felt responsible for patients, whose calls they replied. They could only feel released from this responsibility by getting an appointment to a physician for the patient, which in some cases took a few days. According to the nurses, this led to a competition to get appointments to their own patients as quickly as possible. However, at a primary care centre the nurses decided to change their way of working and prioritising, in order to reduce this stress. They introduced a new distribution process, which was based on a shared list that included all those patients waiting for an appointment. Then the nurses worked together to allocate available doctor visits to patients according to their need for care.

### Technical systems

Technical advancement and the county councils procurance of technical systems, created both possibilities and limitations for how situations with limited resources could be handled.

According to the informants, the primary care centre’s overall organisation, as well as any technical advancements in systems used for appointment booking or handling phone-calls, to some extent predetermined the variation in patient influx. For example, the work-schedule was only available for approximately eight hours a day during weekdays, thus the influx of patients had to fit within these hours. Furthermore, the informants explained that all appointments were pre- marked in the digital schedule with either 15- or 30-min slots and that there were mostly these options to choose from, when distributing appointments.

“The whole principle with web-based appointments where patients are entitled to a part of the publicly financed health care system, which there is not much of, but everybody wants, without medical prioritisation, I consider it a crime, it is against the Swedish Medical Service cornerstone’s Act, which stipulates that care should be distributed according to need. There will not be care according to need, there will be care to those who are digitally knowledgeable and find this and can book themselves, and the old and digitally unknowable are mainly left behind, maybe the poor too, who do not even own a computer, they are left behind or outside. Yes, I am very upset over this, as you can see” -Physician 2

Patients’ preferability for choosing which entry point to use when contacting their primary care centre, lay within the technical systems feasibility. The informants problematised how technical advancements had offered patients freedom to book appointments directly to a physician. However, this service was described to not only undermined technically weak patients’ possibility to get health care, but it also impacted the informant’s possibility to distribute health care resources to those who are worse off.

### Organisational culture

Workplace culture was described as both normalising and restricting, for how some situations were handled. The informants explained they maintained bad work-culture and risked their well-being when unreflectingly resolving large workload by working late.

“Just that somebody pops by and diverges [from work] “right, I have a break/ but is it just me, then I’m not aware that I could do that. But it is more, that it looks the same in my colleague’s room [they are also not taking a break], so I think that it matters, what type of culture, if you call it that, what sort of “break culture” a workplace has” -Physician 1

The informants stressed that colleagues’ behaviour that lay outside the local workplace-culture affected them to adapt. Additionally, a workplace culture that did not promote professionals to listen to each other’s views, had a negative influence on how consensus was formulated, and situations resolved.

## Actions

The informants performed different actions to handle situations with limited resources. What, when and how many actions were performed at a certain time, depended on the prevailing combination of patient influx and structural conditions.

### Matching health care needs with professionals’ competency

Successfully matching health care needs with the corresponding competence of a professional, was described as especially important for patients with greater health care needs. An experienced physician was more effective during their disposable time, and performed more tests/examinations, which implied that booking additional appointments were not necessary.

“ If you have a complex [health care need] where there are difficult and many things, then as the first option I would rather not book them to an intern, and if that is done then maybe you have to talk with the intern so they consider it okay. Otherwise, if they do not have enough competence, it will be wrong for both the patient and physician,” -Nurse 5

Furthermore, the informants explained they did not consider it fair to expect of interns during their medical residency, to handle great health care need with the same capacity as a physician. Moreover, it was described as good for the patients as they received what they needed, and did not have extra visits, but also for the centre, which used its resources in an appropriate way.

### Categorising health care needs to suit appointment booking systems

As the length of the appointments were pre-determined by technical systems, patient’s health issue was divided to fit the available time slots.

“so, it is a little bit different how it is, sometimes you have to divide them, or more often the urgent and the chronical, long-lasting problems are divided” -Nurse 1

The informants explained that appointments with a shorter duration (15 min) were meant for “minor” health care needs that could be addressed during that time, such as tonsillitis, pneumonia, or a sore throat. Appointments with a longer duration (30 min) were meant for greater health care needs, such as stomach pain, which needed more time to examine and treat. If there was no duration that fitted the patients’ issue near in time, patients were booked for two separate appointments, occasionally with weeks in between.

An example of this was a patient who contacted the centre for a small cut, received a short visit the same day. However, the patient also described unexplained weight loss and fatigue for which an extended visit in a couple of weeks was booked.

### Giving care at the inappropriate health care level

Another example of handling the miss-match of patient influx and available competencies was to knowingly assign care to the inappropriate health care level.

“However, if it is prioritised that a physician appointment is needed, then it is important that you get it. And that prioritisation must be managed in the longest, but if there are no resources left at the primary care, then you must redirect patients to the emergency care. We also have limited resources regarding time and physicians’ appointments, and those we really need to prioritise, that is how it is” -Nurse 4

The interviewed nurses explained that they felt forced to only give self-care- advice and urge patients to seek care if their condition worsened when there were no appointments available in the near future. The informants explained that they were *“out on thin ice”* (Nurse 5), as this advice risks undermining patient safety. Another approach was to forward patients to the emergency room, even though the informants thought the appropriate level of care was the primary care. They also used the wrong level of care within the centre as patients were knowingly booked to the inappropriate profession. For example, a patient with a sudden onset of vertigo was booked to a nurse with the intention of excluding something more serious with an electrocardiogram (ECG). However, the interviewed nurses acknowledged, that there were risks associated with becoming a *“mini physician”(-* Nurse 3) for diseases that are outside their field of expertise.

### Rearranging workhours

Workhours were described as flexible, and the informants used two approaches; shuffling, and maximising, to get the best use of their time.

“it has more to do with what do I have in front of me right now, the appointment with the patient I just had, because it is really hard to appreciate beforehand when the appointment is booked, how much time will this take… So, if there is something that takes more time, then it is most often the breaks that are at risk to be shorter, or to vanish, it is the same with the lunches, so…” -Physician 1

Shuffling workhours included actions that prolonged their workday, such as overbooking their timebook or working during breaks. Most administrative tasks related to a patient appointment, were instantly solved if the informants did not take a break. However, the informants expressed worries about working over breaks, as it could potentially compromise their long-term health, and well-being. Redistributing more work hours, to clinical work was done by postponing administrative tasks or refraining from further training courses. One physician placed work hours outside the primary care centre’s opening hours to work undisturbed and finishing administrative tasks that piled up or were set aside, in favour of questions from colleagues or patients.

“If there is somebody that is at the laboratory [having their blood drawn] and you have two minutes left while waiting, then you can sign [medical journals], but it is time that does not really exist. Sometimes you have multiple activities going on at the same time, if you take an EKG, and you go to the laboratory, and the third does something else, and then you can get some breathing space, and then you can sign, or as I said, go through answers from incoming blood work, maybe you fit in an phone call…or get an message from a nurse that “this one wants to come in contact”…”-Physician 6

Maximising workhours included actions that utilised the informants’ or their colleagues’ time, in the best way possible. When there was a discrepancy between available appointments and the influx of patients seeking care, the informants described they did not ask extensive questions about the patients’ issue; instead, they worked quickly and booked the patients to the appointment they needed. Furthermore, the informants described using their workhours effectively by eliminating idle time as they performed administrative tasks and answered phone calls, all while waiting for patients or lab results.

According to the informants, the maximising of workhours was also beneficial for patients, if they did a thorough job. One physician explained that skin rashes usually *"snuck in"* (Physician 2) at the end of other appointments and were managed by taking a quick look. If the rash was benign, nothing more was done. Moreover, if a rash was malign, all necessary measures were taken as patients were quickly sent to a dermatologist instead of waiting for a new appointment. Also, patients could be prevented from calling the primary care phone line if the informants were not careless, and if they finished any patients related tasks adjacent to an appointment, such as writing journal notes, medical receipts, or certificates. Furthermore, extra phone calls were also avoided when they did a right assessment through the phone and forwarded the patient to a professional most suitable to examine their issue, like a physiotherapist.

“then there is one clear as a bell [that the patients must have an appointment today], that you have to puzzle, then you find fifteen minutes and you know”well, I will be late to the other patients”, but you simply squeeze it in, you trick and fix. If you need to put it on another colleague, overbook a colleague, then I of course talk with that individual beforehand, but that’s how it is”- Nurse 2

The informants were conscious about the centres resources and explained how they tried to maximise the use of their colleague’s workhours as well. One approach was to use the natural course of a disease and ask patients to call the following day, hoping their health had improved enough not needing an appointment. Other approaches were prolonging durations between two-revisits or rebooking patients for another day or with a colleague. On rare occasions appointments were cancelled, and not re-booked. However, the informants emphasised that cancelling appointments was only used for patients with a non-urgent health care need in favour of those with a more urgent one.

### Passing on the decision making

Situations that could not be solved by the informants were referred to the primary care manager, a professional with a higher competence or a colleague deemed responsible for making decisions concerning how to proceed and prioritise. Hence, it spared them from being responsible for solving a situation caused by limited resources.

“No, this, this is not included in my job description, to get this primary care to go together, I have a more limited assignment than that” -Physician 1

The extent to which the situation was resolved was limited by position and profession.

For example, an interview nurse described that after consulting a physician about a patient, it was deemed that the patient must be looked at the same day. Since, there were no appointments left for that day, the decision for how to solve the situation was passed on to the primary care manager. The informants emphasized that they did not have infinite responsibility to solve a problem that arose due to limited resources. They needed to constantly remember that they could pass on these types of decisions to a person with greater responsibility or competence. If such decision was delayed, the informants still tried to do something for the patient while they were waiting.

Sometimes uncontroversial actions, such as renewing prescriptions for a colleague’s patients, could become controversial if one did not have the same collegial view, for example, when narcotic-classified drugs should be prescribed. At one centre, the physicians describe they made a collective decision to only renew the minimum amount of narcotic drugs to ensure that patients would make it until their ordinary physician was back at work, and take further decisions concerning for how to proceed. If this type of prescription was not renewed, it only led to patients calling or visiting the primary care centre in despair, which burdened other professional groups.

## Discussion

This study contributes with knowledge on how situations with limited resources within primary care are handled by nurses and physicians. The result demonstrates that the organisation’s structural conditions are important, for the repertoire of different actions that the health care professionals create and use in their endeavour to meet the influx of patient’s health care needs within limited resources. It shows their creativity in adapting these actions in their pursuit of good care and equal health in the population. The findings elucidate the importance of understanding the context within which priorities take place and that professionals’ actions and priorities are situated.

The present study is conducted in a Swedish health care context, which is ruled by three ethical principles for how resources should be prioritised and has as its main goal to achieve equal and good health for the entire population. These findings reflect both the wide responsibilities that primary care have, and the important role that the human dignity principle has, and its relevancy within primary care. For instance, citizens with various health related problems are handled and, even those that are not suitable at a primary care level receive help by being referred to other health care services or institutions.

Our findings indicate how the need and solidarity principle is somehow undermined by structural conditions in primary care. For example, higher authorities have decided the conditions for primary care, such as goals for continuity, maximum waiting time guarantees and the procurement of technical systems. These conditions govern both what professionals must, and what they can do, which may conflict with their ability to comply with the need and solidarity principle.

In other research, economic incentives have been shown to affect how primary care organised accessibility to care, which leads to minor health care needs being prioritised at the expense of those with greater health care needs. Our findings complement previous studies [15, 30, 31] by highlighting how policies and digitisation, through technological advances have restricted professionals’ ability to ration care and de-prioritise patients with minor health care needs. Moreover, that technological advances such as self-booking services, create actions that risk discriminating against less digitally knowledgeable patients. This implies that attention must be given to how primary care professionals can comply with the need and solidarity principle when implementing decisions, reforms or digital systems that affect the context in which professionals act. As shown by our study, prioritisations in primary care cannot be decontextualised.

Our findings suggest that the staff, through their actions, strive to respond to the principle of need and solidarity and as far as we can shed light on this through our study, to some extent also the principle of cost-effectiveness. For example, patients who had poorer health received more resources, either by having more visiting appointments or higher competence in the care staff, both of which are associated with higher costs. This emphasise the importance of considering the efficiency of how the use of different professionals’ competencies is organised and carried out, to make the best use of resources and carry out cost-effective care measures at a reasonable cost, in the pursuit of good and equal health in the population.

As we can see by the findings, the staff must constantly make different choices and decisions to make limited resources fit with the influx of healthcare needs. This corresponds well with what is described in the garbage can model [32], which highlights that actions in a situation do not necessarily start with a problem looking for a solution, but rather actions looking for a situation to which they might be the answer, and professionals looking for something to do. Although the actions in our findings were created and paired with a situation they were proposed to solve, it was not always the best action performed, neither for the professional nor for the patient. As health care professionals knowingly assigned patients to care at the inappropriate health care level, worked over their breaks, and passed on responsibility, it indicates that they would rather do something, than nothing to solve a situation. Furthermore, this also highlights that prioritising in primary care is highly contextual and situated. Our findings also complement previous studies on the importance of organisational culture [33, 34] by adding that a work- culture which promotes a healthy work-life balance is required for primary care professionals to create actions that do not compromise their own well-being.

However, the action passing on the decision making, highlights the ambiguity described in other studies regarding who has the ultimate responsibility for prioritising [9, 25, 35] This demonstrates the importance of future studies examining politicians' discretion to govern primary care, and managers conditions to allocate limited resources to achieve good and equal health in the population.

### Methodological considerations

By using purposive sampling, we were able to include participants with different experiences due to the centre’s size and geographical location. Purposive sampling is an appropriate strategy when needing to find informants that can provide in-depth and detailed information about a subject. Due to a recorder malfunction, the last interview was not recorded. Therefore, notes written down from the interviewer’s memory, together with field notes, were included in the analysis. We judge the loss of data as minor as we had fourteen interviews altogether and data saturation seems to occur within the first twelve interviews [36].

The study was conducted with a limited number of health care professionals from a single region in Sweden. To enhance the understanding of the transferability of the findings, we have provided information on the context in which the study was performed, described the recruitment study participants, interview questions, and analysis procedure as thoroughly as possible [26, 37].

To improve the credibility of the work, informants with different medical backgrounds, educational levels, and working experience from health care and primary health care were included. Furthermore, all the authors involved in the analysis had different competences. The first author is an PhD student with medical educational background and experience from working clinically in primary care. The first author’s previous experience may have an impact on how follow-up questions were asked during the interviews. Prior knowledge about the specific primary care centres where informants work could bias the analysis. However, to achieve neutrality in the interviews and analysis, there were few interactions with the centre managers prior and post the interviews.

To enhance confirmability, categories found in the analysis were reviewed in relation to the entire data, and illustrative extracts have been provided. Also, regular analytical sessions were initially held between the first and fourth author, whereas the whole research team was included in the analytical sessions when there was a first draft.

### Implications

This study implies that:

- attention must be given to the context in which health care professional make prioritisations, to facilitate prioritisations contributing to equal health in the populations.
- Collegial agreement for how to handle certain situations can be a helpful starting point for reflections regarding who, what, how, why, when, and where patients are prioritised.
- In order to better understand the importance of the context for priority setting and rationing, studies should examine politicians' and operational managers' views on their own discretion to govern and allocate resources, and to manage available resources, respectively.

## Conclusion

Priorities in primary care are not, “one fits all” solution. Our study shows that priorities in primary care comprise of ongoing daily processes that are adapted to the situation, context of patient influx, and structural conditions. Healthcare professional’s actions for how influx of patients’ is handled in relation to limited resources, are created, and shaped within this context which also sets the boundaries for their actions.

## Data Availability

The interviews generated analysed during the current study are not publicly available due risk of identifying the informants but are available from the corresponding author on reasonable request.
